# Bringing into focus the central domains C3-C6 of myosin binding protein C

**DOI:** 10.3389/fphys.2024.1370539

**Published:** 2024-02-29

**Authors:** Chang Yoon Doh, Alexandra V. Schmidt, Krishna Chinthalapudi, Julian E. Stelzer

**Affiliations:** ^1^ Department of Medicine, School of Medicine, Case Western Reserve University, Cleveland, OH, United States; ^2^ Department of Physiology and Biophysics, School of Medicine, Case Western Reserve University, Cleveland, OH, United States; ^3^ Department of Physiology and Cell Biology, Dorothy M. Davis Heart & Lung Research Institute, College of Medicine, The Ohio State University, Columbus, OH, United States

**Keywords:** myosin binding protein C, central domains, hypertrophic cardiomyopathy (HCM), muscle regulation, protein dynamics and conformation, protein interactions, structure and function, post-translational modifications (PTM)

## Abstract

Myosin binding protein C (MyBPC) is a multi-domain protein with each region having a distinct functional role in muscle contraction. The central domains of MyBPC have often been overlooked due to their unclear roles. However, recent research shows promise in understanding their potential structural and regulatory functions. Understanding the central region of MyBPC is important because it may have specialized function that can be used as drug targets or for disease-specific therapies. In this review, we provide a brief overview of the evolution of our understanding of the central domains of MyBPC in regard to its domain structures, arrangement and dynamics, interaction partners, hypothesized functions, disease-causing mutations, and post-translational modifications. We highlight key research studies that have helped advance our understanding of the central region. Lastly, we discuss gaps in our current understanding and potential avenues to further research and discovery.

## Introduction

Myosin binding protein C (MyBPC) is an essential regulator of contractile function in skeletal and cardiac muscle (Bennett et al., 1999; Oakley et al., 2004; Heling et al., 2020; Song et al., 2023). Initially, MyBPC was visualized as an unknown thick filament structure in frog sartorius muscle via low-angle X-ray diffraction experiments and stripes of extra mass 43 nm apart in long sections of muscle (Huxley, 1967; Huxley and Brown, 1967). MyBPC, or “C-protein” as it was called initially, was discovered a few years later and was characterized as a long rod-shaped, thick-filament associated protein weighing about 140 kDa with little alpha helix content (Starr and Offer, 1971; Offer et al., 1973). Subsequent studies confirmed that MyBPC localized to the A band of thick filaments with approximately 43 nm spacing (Hanson et al., 1971; Rome et al., 1973; Pepe and Drucker, 1975). Early biochemical assays showed that MyBPC had at least three different paralogs: slow skeletal, fast skeletal, and cardiac (Callaway and Bechtel, 1981; Reinach et al., 1982; Yamamoto and Moos, 1983; Kawashima et al., 1986).

**FIGURE 1:**
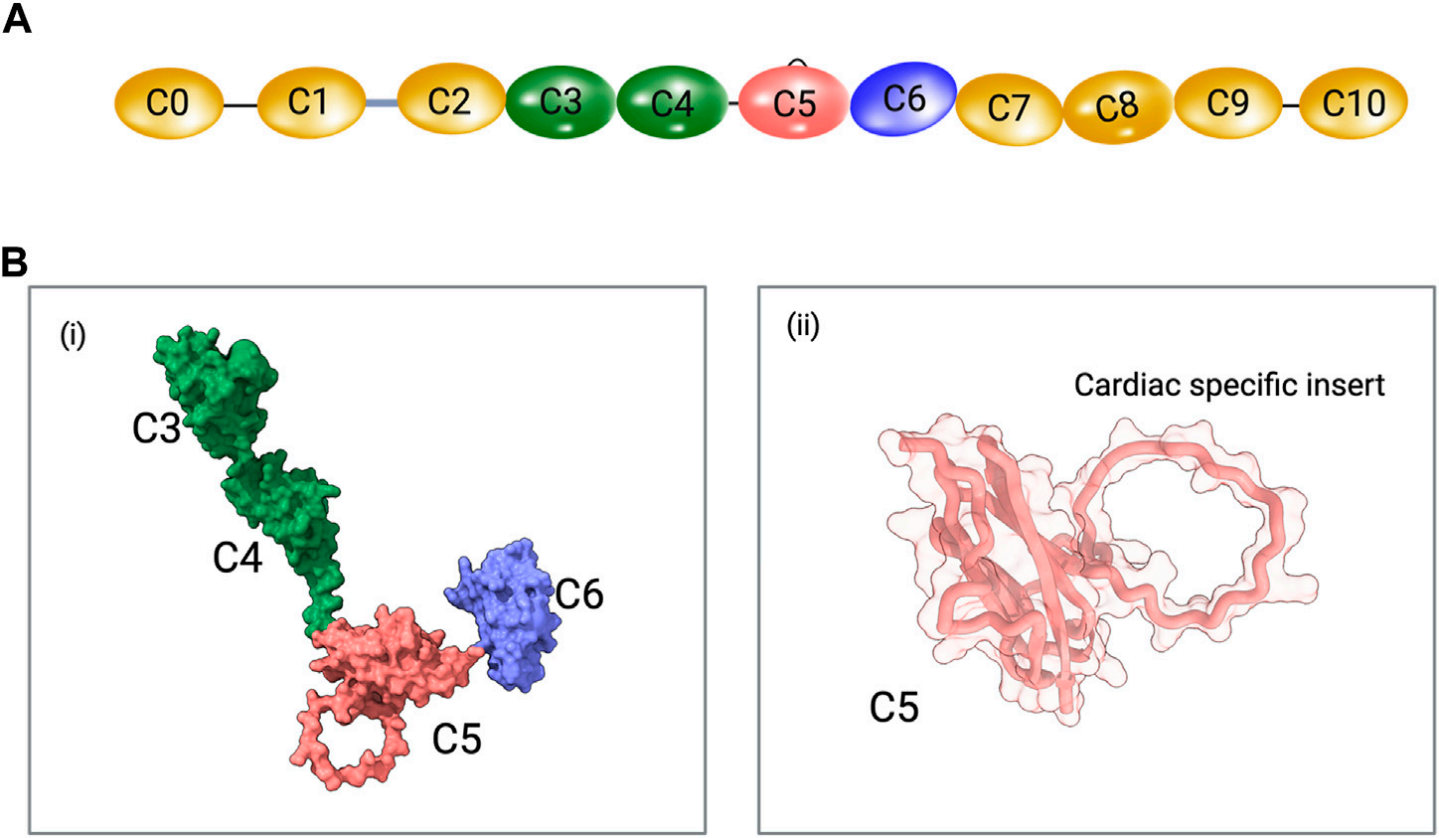
**(A)** Schematic representation of cardiac MyBPC domains. The C0 and C1 domains are connected by a proline-alanine rich (P/A) linker and the C1 and C2 domains are connected by the MyBPC motif (M-domain). The cardiac-specific C5 domain insert is shown. Schematics are not drawn to scale. **(B)** i. A predicted model of C3-C6 domains of MyBPC using AlphaFold. ii. The cardiac C5 domain with cardiac-specific insert is shown.

With the development of modern gene sequencing and molecular cloning techniques, it became possible to study MyBPC in a more detailed manner. Each paralog of MyBPC was classified as a multi-domain protein containing immunoglobulin-like domains of the C2 type and fibronectin type 3 domains (Einheber and Fischman, 1990; Fürst et al., 1992; Weber et al., 1993; Gautel et al., 1995) (Figure 1A). This led to extensive domain-specific studies of thick filament assembly and interactions with myosin and titin involving domains C7-C10 (Okagaki et al., 1993; Freiburg and Gautel, 1996; Gilbert et al., 1996), as well as studies of functional and regulatory roles of the N-terminal C0-C2 domains regarding actomyosin interactions and regulation of crossbridge function (Gruen and Gautel, 1999; Harris et al., 2004; Razumova et al., 2008; Risi et al., 2018). The central region of MyBPC has not been the focus of the majority of research, and the potential functional and regulatory roles of domains C3-C6 are largely unknown.

The complete sequencing of the human cardiac MyBPC gene (*MYBPC3*) paved the way for domain-specific studies (Carrier et al., 1997). The *MYBPC3* gene is >21,000 base pairs in size and contains a total of 35 exons (Carrier et al., 1997). The central domains were translated from the following exons: exons 16–17 (C3 domain), exons 18–19 (C4 domain), exons 21–23 (C5 domain), and exons 24–25 (C6 domain). Exon 20 encoded the C4-C5 linker (VKIDFVPRE) and exon 22 approximately coincided with the loop region (GNKAPARPAPDAPEDTGDSDEWVFDKK), which are sequences specific to the fast skeletal/cardiac and cardiac muscle paralogs, respectively (Carrier et al., 1997). Because different paralogs were formed from different combinations of domains, it was hypothesized from early on that these paralog-specific domains may have a role in the function and regulation of different MyBPC paralogs (Hartzell and Glass, 1984; Hartzell and Sale, 1985; Schlender and Bean, 1991; Gautel et al., 1995).

In addition, *MYBPC3* was found to be linked with familial hypertrophic cardiomyopathy (HCM) (Carrier et al., 1993; Watkins et al., 1995; Maron and Maron, 2013). HCM has been thought to be a common cause of sudden cardiac death (SCD) in younger individuals, but more recent studies have shown that the rates of SCD in HCM patients are closer to aged-matched general population (Saberi and Day, 2018; Weissler-Snir et al., 2019). Of all of variants of the *MYBPC3* gene, –64% are missense variants, –30% are synonymous variants, –3% are frameshift and truncation variants, and 3% are in-frame indels and splice site variants (Harris et al., 2011; Helms et al., 2020; Desai et al., 2022). Some of these variants are pathogenic or likely pathogenic, while others are not (Doh et al., 2023). A query of the Sarcomeric Human Cardiomyopathy Registry (SHaRe) showed that disease-causing mutations in *MYBPC3* are the most common cause of familial HCM (Helms et al., 2020), with the vast majority of these variants within the central domains (Suay-Corredera et al., 2021a). This suggests that the central region has a function beyond that of a spacer between the C- and the N-termini.

Since then, there has been little progress in furthering our understanding of the central C3-C6 domains of MyBPC. Therefore, we aim to summarize the current views and hypotheses regarding the domain structures, arrangement and dynamics, interaction partners, hypothesized functions, disease-causing mutations, and post-translational modifications (PTM) in the C3-C6 domains of MyBPC.

## Structures of the central domains of MyBPC

Cardiac MyBPC is a key regulatory sarcomeric protein in the heart muscle, playing a critical role in cardiac muscle contraction. MyBPC is located in the C-zone of the A-band, where it interacts with both myosin and actin filaments (Heling et al., 2020). MyBPC consists of multiple immunoglobulin (Ig) and fibronectin type-III (FnIII) domains, which contribute to its modular or flexible structure (Ackermann and Kontrogianni-Konstantopoulos, 2011). Notably, the N-terminal region of cardiac MyBPC contains phosphorylation sites that are essential for modulating cardiac function, especially during sympathetic stimulation where it enhances heart rate and force of contraction (Barefield and Sadayappan, 2010; Rosas et al., 2015; Mamidi et al., 2016; Ponnam and Kampourakis, 2022). Mutations in *MYBPC3* are linked to various cardiomyopathies, underlining its importance in cardiac physiology and clinical cardiology (Helms et al., 2020; Tudurachi et al., 2023). The ability of cardiac MyBPC to bind and stabilize myosin filaments, as well as its interaction with actin, positions it as a key regulator of sarcomeric structure and, consequently, cardiac muscle function. Key to this understanding is the identification of specific domains that bind to either myosin, actin, or other sarcomeric proteins.

MyBPC is a multidomain scaffolding protein and its domains are arranged in a complex manner to facilitate muscle contraction. The structural details of MyBPC domains are revealed by techniques such as X-ray crystallography, nuclear magnetic resonance (NMR), and cryo-electron microscopy (cryo-EM). The human cardiac MyBPC C5 domain was the first domain solved using NMR in 2003 (PDB ID: 1GXE) (Idowu et al., 2003). Structural analysis of the C5 domain revealed a beta-bulge structure at the N-terminus facilitating the C4 and C5 domains in close proximity. A 28 amino acid “CD” strand loop was revealed to be unique to the C5 Ig domain of cardiac MyBPC and was also absent in the non-cardiac MyBPC paralogs. (Cecconi et al., 2008; Guardiani et al., 2008). The increased mobility and flexibility of the C5 domain were predicted based on sedimentation and ^15^N relaxation experiments (Idowu et al., 2003).

Four solution and crystal structures of the C3 domain have been published: human slow skeletal MyBPC (PDB ID: 1X44; NMR), human fast skeletal MyBPC (PDB ID: 2EDK; NMR), human cardiac MyBPC (PDB ID: 2MQ0; NMR), and human cardiac MyBPC with the R502W substitution (PDB ID: 2MQ3; NMR) (Zhang et al., 2014). The C4 domain structure was solved using NMR experiments from the human slow skeletal paralog (PDB ID: 2YUZ) and mouse fast skeletal paralog (PDB ID: 2DLT). There is no known solved experimental structure of the C6 domain. With the recent advancements in *in silico* structure prediction and modeling programs, it is possible to predict all eleven domains of MyBPC based on amino acid sequence using tools like AlphaFold (Jumper et al., 2021; Zhou et al., 2022b) (Figure 1B).

## Arrangement and dynamics of the central domains of MyBPC

Early *in vitro* binding assays showed that the C-terminal end of MyBPC is bound to the myosin thick filament and interacts with titin (Starr and Offer, 1978; Okagaki et al., 1993; Freiburg and Gautel, 1996; Gruen and Gautel, 1999). However, the exact configuration of MyBPC on the thick filament remains unclear. There have been many theories regarding the arrangement of MyBPC on the myosin filaments, reviewed in detail elsewhere (Bennett et al., 1999). Some of the proposed configurations of MyBPC on the thick filament have included 1) axial arrangements of myosin and MyBPC in a vernier mechanism (Huxley and Brown, 1967), 2) repeats of three molecules of MyBPC wrapping themselves around the thick filament to form a “trimeric collar” (Swan and Fischman, 1986; Flashman et al., 2008), or 3) MyBPC molecules forming lateral or radial projections from the thick to the thin filaments (Moos et al., 1978; Squire et al., 2003).

Several EM and X-ray fiber diffraction studies generated low-resolution 3D structures of the human cardiac filaments-uncovering the positioning and potential function of cardiac MyBPC in relation to the thick (Zoghbi et al., 2008; AL-Khayat et al., 2013; Nag et al., 2017; Tamborrini et al., 2023) and thin filaments (Luther et al., 2011; Mun et al., 2011; 2014; Risi et al., 2018; Tamborrini et al., 2023). An immuno-EM study showed that antibodies specific to the central domains (C5-C7) labeled the same nine axial positions within the A-band, suggesting that both the N-terminus and central domains of MyBPC run approximately transversely to the thick filament and are oriented radially to the sarcomere (Lee et al., 2015). In 2011, an electron tomography (ET) study showed that MyBPC binds to actin and that it may be in a radial orientation with a bend at the C5 domain (Luther et al., 2011). More recently, the C3-C6 domains were predicted to form a bridge from the thick to the thin filament using AlphaFold generated domains as models in the low-resolution −18 Å cryo-ET composite reconstructions, with high angular distribution relative to the thick filament *Z*-axis, supporting the prior experimental observation (Tamborrini et al., 2023). Additionally, a recent cryo-EM study, at a resolution of 6–10 Å, showed that the central domains and the cardiac-specific 28 amino acid loop insert in the C5 domain have important interactions with various locations on the interacting-heads motif (IHM) of myosin (Dutta et al., 2023). Mavacamten, a small molecule cardiac myosin inhibitor that stabilizes the folded state of myosin, was utilized in these recent studies (Dutta et al., 2023; Tamborrini et al., 2023). While these structural studies are valuable and provide some initial insights into the domain structure and arrangements in the presence of mavacamten in the C-zone, a high-resolution and unambiguous reconstruction of the domains is needed for accurate analysis of inter-domain and inter-molecular interactions.

Additionally, early research involving rotary shadowing techniques showed that fast skeletal and cardiac MyBPC paralogs (containing the elongated C4-C5 linker) adopted various conformations including bent V or U shapes (Hartzell and Sale, 1985; Swan and Fischman, 1986). This ability of MyBPC to bend at domain junctions was reinforced by an EM study showing that MyBPC indeed had internal hinges in the molecule, with one in the M domain and one near the C4 and C5 central domains (Previs et al., 2016). Several studies have shown that MyBPC is a dynamic molecule with internal hinges that permit a wide range of motion (Colson et al., 2016; Previs et al., 2016). Although the N-terminal domains primarily have been shown to influence actin binding and rotational dynamics, it is hypothesized that the central domains’ internal hinge (C4-C5) may also contribute to this action (Colson et al., 2016; Nadvi et al., 2016; Doh et al., 2022a). More recently, a “hinge-and-latch” mechanism was proposed to explain the biophysical properties of this region through molecular dynamics simulations (Doh et al., 2022a). By creating constructs with deletions in the linker and loop regions, the authors showed that the C4-C5 linker aids in reducing the C4 and C5 interdomain angles (acting as a “hinge”), while the C5 loop region helps form sustained interdomain interactions in the presence of the linker region (acting as the “latch”). It is hypothesized that the “hinge-and-latch” mechanism is unique to the cardiac MyBPC paralog, which may contribute to an paralog-specific mechanism (Doh et al., 2022a).

## Interaction partners of the central domains of MyBPC

Initially, MyBPC was found to interact with the myosin rod, light and heavy meromyosin, and myosin subfragment 2 (Moos et al., 1975; 1978; Starr and Offer, 1978; Okagaki et al., 1993; Gruen and Gautel, 1999; Flashman et al., 2007). Further studies revealed that MyBPC also had interactions with titin (Tonino et al., 2019), regulatory light chain (Ratti et al., 2011), actin (Moos et al., 1978; Whitten et al., 2008; Kensler et al., 2011; Luther et al., 2011; Mun et al., 2011; Orlova et al., 2011; Belknap et al., 2014; Risi et al., 2018), calmodulin (Lu et al., 2012), formin homology 2 domain-containing 3 protein (Fhod3) (Matsuyama et al., 2018), and four and a half LIM protein 1 (Fhl1) (McGrath et al., 2006a). Unfortunately, most of these interaction studies utilized the whole or large fragments of MyBPC (McGrath et al., 2006b; Ackermann and Kontrogianni-Konstantopoulos, 2011; McNamara and Sadayappan, 2018).

To determine specific domains that could be interacting with key myofilament proteins, studies using isolated domains were conducted. Early studies using yeast two-hybrid (Y2H) assays showed that domain C5 had a strong affinity for domain C8, confirmed by surface plasmon resonance (Moolman-Smook et al., 2002). Based on their data, the authors hypothesized that MyBPC may form a trimeric collar around the thick filament that would be dynamically formed and released, affecting cross-bridge formation. A follow-up study revealed that this interaction between C5 and C8 is also present in fast skeletal but not slow skeletal paralogs (Flashman et al., 2008). It has also been speculated but never confirmed, that the C5 domain’s cardiac-specific loop may function as a scaffold for various signal transduction molecules containing SRC (short for sarcoma) homology 3 domains (Gautel et al., 1995).

**FIGURE 2:**
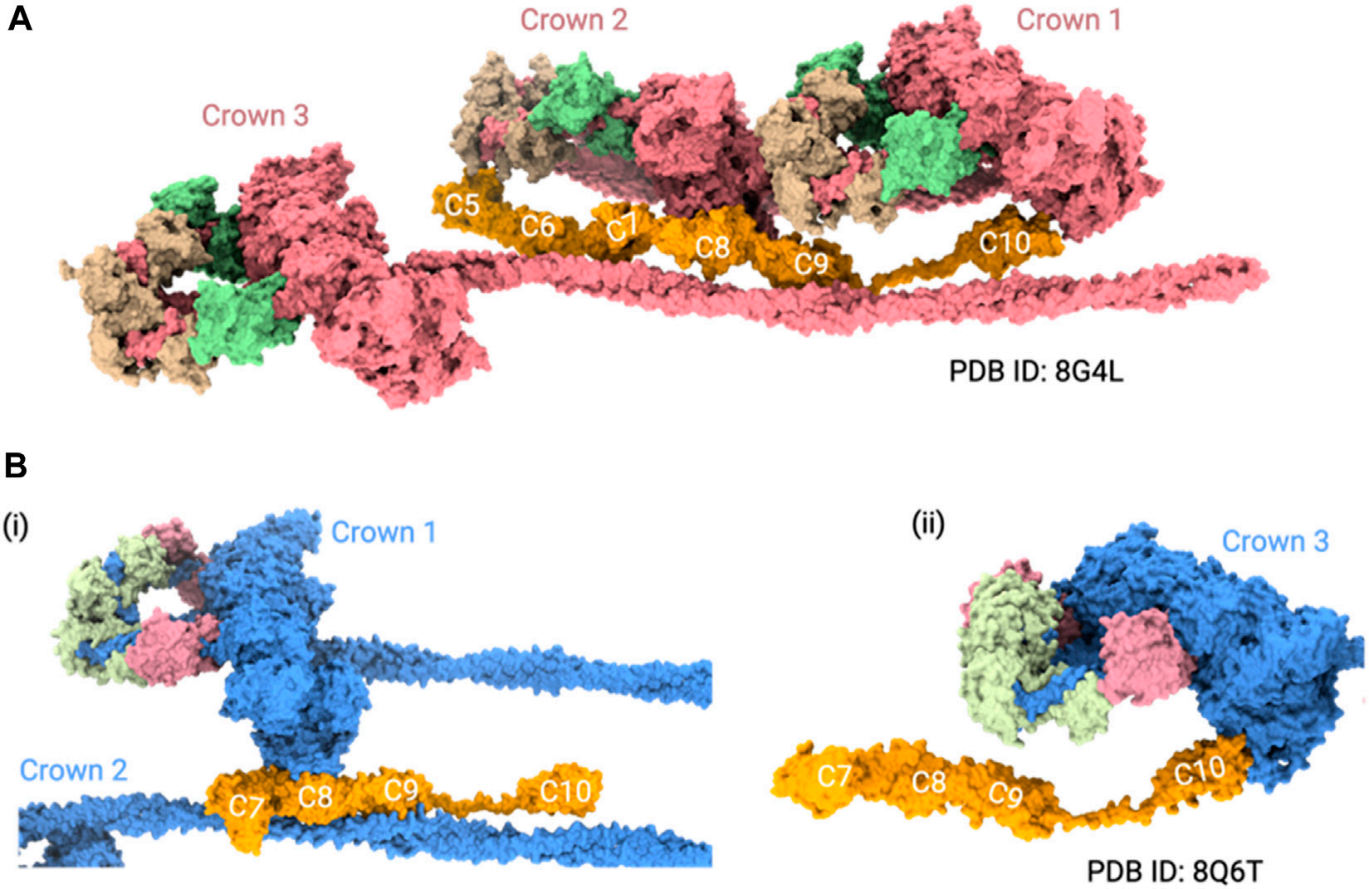
MyBPC central region positioning in thick filament structures. **(A)** Cryo-EM structure (PDB ID: 8G4L) shows the positioning of the C5 to C10 domains of MyBPC in the thick filament. In this model, the C5, C8, and C10 domains of MyBPC interact with IHM. **(B)** Cryo-ET structure (PDB ID: 8Q6T) shows a mouse thick filament structure with the rigid body docked, AlphaFold predicted C7-C10 domains of MyBPC. i. The C8 domain interacts with one of the crowns and ii. the C10 domain of MyBPC interacts with another crown. For clarity of visualization, we only showed three crowns of thick filament in both structures.

Experiments involving truncated recombinant proteins generated through the baculovirus/insect cell system showed that C5-C9 domains were required for binding to beta-myosin heavy chain (Flavigny et al., 2003). Although this study did not clarify which exact region had a role in myosin binding, it narrowed down the potential interacting domains. A more recent microscale thermophoresis (MST) experiment showed that the highest affinity sites on MyBPC for myosin S1 are not located in the N-terminus, as previously thought, but within the central regions (Ponnam and Kampourakis, 2022). They showed that C2-C4 and C5-C7 segments compete for the same binding site on myosin S1, suggesting that MyBPC crosslinks to S1 and S2 of a single myosin molecule, resulting in increased stabilization of myosin in the folded state (Ponnam and Kampourakis, 2022). They also showed that these peptides interacted with mini-HMM fragments (a combination of myosin S1 and S2 fragment neck region) and the bare S2 tail (Ponnam and Kampourakis, 2022)**.** Interestingly, the positioning of the central domains in recent cryo-EM studies is ambiguous in the mavacamten treated thick filaments (Dutta et al., 2023; Tamborrini et al., 2023). The cryo-EM study showed the C5-C10 domains in their reconstructions, and proposed that C5, C8, and C10 domains interacted with the IHM. Specifically, it predicted that the 28 amino acids in the cardiac paralog-specific insert of the C5 domain may interact with CrH (crown containing horizontal IHM), and the central domains may also interact with TaH and TaT (tails originating from tilted and disordered IHM) in the folded-back state (Dutta et al., 2023) (Figure 2A). However, the cryo-ET study showed the C7-C10 domains in their reconstructions, and they showed that the C8 and C10 domains interacted with IHM, but the C5-C6 domains were not resolved (Tamborrini et al., 2023) (Figure 2B). The common feature between these structures was that the C8 and C10 domains interacted with the IHM.

While these studies advanced our understanding of thick filaments with mavacamten, the heterogeneity of MyBPC interactions remains apparent. For instance, it is ambiguous whether the MyBPC hinge is near the C7 domain or the C5 domain based on the cryo-EM/cryo-ET reconstructions (Dutta et al., 2023; Tamborrini et al., 2023). In addition, more studies are necessary to determine how the central region interacts with myosin filaments when some of the myosin heads form IHM structures and others do not form, much like in the native thick filaments (Nag and Trivedi, 2021; Walklate et al., 2022).

## The hypothesized function of the central domains of MyBPC

Despite early studies that established MyBPC’s functional role in actin-activated ATPase activity and contractile function of skeletal and cardiac myosin (Offer et al., 1973; Moos and Feng, 1980; Hartzell, 1985; Hofmann et al., 1991; Weisberg and Winegrad, 1996), the role of the central domains in contractile function and regulation has only been of recent interest. Although there were hints of the central domain’s role in regulating contractile function, there were no clear explanations for many years (Heling et al., 2020).

Given the relatively high conservation of amino acid sequence across various regions of the central domains, they may have evolutionarily important regulatory or functional roles across species (Oakley et al., 2004; Karsai et al., 2013; Doh et al., 2022a). One study aimed to clarify the role of central domains in calcium-activated force generation. Isolated skinned rat cardiac trabeculae were incubated with C5, C2-C5, or C2-C4 to interfere with interactions of the endogenous C5 domain (McClellan et al., 2004). C5 and C2-C5 peptide fragments at concentrations ranging from 2–16 µM reversibly reduced Ca^2+^ sensitivity compared to the control C2-C4 fragments, indicating a C5-specific effect on calcium sensitivity (McClellan et al., 2004). The authors also noted an irreversible reduction in F_max_ (Ca^2+^-activated maximal force measured at pCa 4.5) observed at >10 µM of C5 with a coincident increase in the loss of MyBPC, indicating that the endogenous action of C5 may be to help stabilize and maintain MyBPC on the thick filament (McClellan et al., 2004).

It has been known that myosin S1 interacts with the proximal S2 tail of the same myosin, termed the IHM (Wendt et al., 2001; Heissler et al., 2021). However, it wasn’t until 2017, when a homology model based on a previous low resolution EM map showed that the central and C-terminal domains may help myosin form a sequestered-complex termed the “blocked head,” supported by ATPase assays and MST experiments (Winkelmann et al., 2015; Nag et al., 2017). Specifically, the authors suggested that there may be interactions between the C5 and C7 domains with the mesa of the blocked head and proximal S2 tail (Nag et al., 2017). From these experiments, it is plausible that the central domains of MyBPC help keep the sarcomere in a sequestered state to modulate force generation and contractility. By extension, various alterations such as truncation mutations leading to haploinsufficiency, mutations leading to altered surface interactions, or PTMs changing interaction strength may cause a shift in the IHM to a more active “open head” conformation, resulting in hypercontractility.

The folded back, sequestered IHM structural motif has been ascribed to the super-relaxed state (SRX) physiologically (Stewart et al., 2010; Hooijman et al., 2011; Alamo et al., 2016; Nogara et al., 2016; Anderson et al., 2018a), although more recent single ATP turnover experiences showed that IHM may not be necessary for the SRX state (Anderson et al., 2018b; Rohde et al., 2018; Gollapudi et al., 2021). Nevertheless, the central domain of MyBPC seem to have a role in the SRX state. First, it was shown that the complete ablation of MyBPC destabilized and reduced the proportion of myosin heads in the SRX state (McNamara et al., 2017). An MST binding assay between 25-hep HMM and C0-C7 peptides clarified that the N-terminal and central domains led to an increase in the population of SRX with a resultant decrease in basal ATP turnover rate (Sarkar et al., 2020). When the N-terminal fragment of MyBPC was deleted, there was no impact on the SRX state (Lynch et al., 2021), implying that the central domain may play a role in stabilizing the SRX. Interestingly, super-resolution fluorescence microscopy and stochastic optical reconstruction microscopy imaging results showed that the stabilization of IHM may result from myosin’s interactions with the more internal domains (C4-C6), given the low number of N-termini bound to the myosin filament surface in the SRX state (Rahmanseresht et al., 2021). The high affinity binding between the central domains of MyBPC and myosin S1 (Ponnam and Kampourakis, 2022) thus indicated that the central domains could have had a significant role in shifting the myosin population to the SRX state and modulating the N-terminal dynamics. Finally, recent mechanical experiments on myocardium isolated from hearts of cardiac MyBPC ablated mice following viral transduction of the C3-C10 construct showed improvements in systolic cardiac function compared to sham controls likely modulated by the reduction in the rate of crossbridge recruitment (k_df_) (Merkulov et al., 2012; Li et al., 2020; Dominic et al., 2023), which is dependent on the ratio of myosin heads in SRX vs. disordered-relaxed states (DRX). The structural interactions between the central domains and myosin in the SRX state may underlie these observations.

Although the precise role of the central domain still needs to be confirmed by additional experiments, there is now more evidence of its importance in the structural, dynamic, and functional influence over the contractile apparatus.

## Disease-causing mutations located in the central domains of MyBPC

Most HCM-causing mutations within *MYBPC3* are missense mutations that are expected to incorporate into the sarcomere, suggesting that these domains have a direct effect on regulatory function (Flashman et al., 2004; Helms et al., 2020; Thompson et al., 2021). Surprisingly, the central domains C3, C5, and C6 are among the most highly mutated regions in *MYBPC3* and are hotspots for pathogenic HCM variants (Desai et al., 2022). Truncating mutations (nonsense variants, indels, and splicing) or aberrations in nonsense-mediated mRNA decay can also cause disease through mechanisms of haploinsufficiency or poison-peptides; however, these complex changes are reviewed elsewhere (Carrier et al., 2010; Glazier et al., 2019; Suay-Corredera and Alegre-Cebollada, 2022). Since there are hundreds of articles identifying novel substitutions in cardiac MyBPC, it is not possible to mention all in this brief review; instead, we highlight a few examples of missense mutations that offer mechanistic insights into the central domain’s overall function and regulation.

Since most missense mutations lead to stably incorporated full length proteins, the alterations in structure through amino acid substitutions directly affect ligand binding or function. An NMR study of the C3 domain with an HCM-causing R502W substitution showed altered electrostatic properties without structural or functional abnormalities, suspected to cause disease through disruptions in interactions between sarcomeric partners (Zhang et al., 2014). Other HCM-causing pathogenic substitutions (R654H and N755K in the C5 domain) were shown to influence its affinity with the C8 domain via Y2H assays (Moolman-Smook et al., 2002). Denaturation experiments of R654H and N755K substitutions showed that they induced only partial unfolding of the C5 domains, which is thought to mainly affect ligand interactions through altered surface charges leading to the HCM phenotype (Idowu et al., 2003). An MST study showed that C5-C7 peptides containing N755K and R820Q substitutions had a strongly reduced affinity for myosin S2Δ and mildly altered interactions with myosin S1, compared to control (Ponnam and Kampourakis, 2022). Lastly, a recent study of recombinant proteins with HCM-associated variants (E542Q, G596R, N755K and R820Q) spread over domains C3-C6 showed that the first two variants E542Q and G596R increased the affinity for myosin S1 and ATPase activity, with decreased ability to interact with F-actin (Pearce et al., 2023). This study supports prior hypotheses that missense mutations that do not affect structure or degradation can still directly alter ligand bindings and activity.

Additionally, missense mutations can also manifest disease through a degradative mechanism (loss-of-function), rather than an aberrant gain-of-function mechanism. The HCM-causing W792R substitution in the C6 domain caused abnormal contractile velocity and twitch force in murine engineered cardiac tissue (mECT) that was not statistically different from MyBPC-deficient mECTs (Smelter et al., 2018). The full length MyBPC with W792R also underwent rapid cytosolic degradation, leading to decreased expression (Smelter et al., 2018). Taken together, the W792R substitution likely causes disease through a direct functional disturbance combined with haploinsufficiency. A well-conserved HCM-causing variant in the C6 domain of MyBPC in ragdoll cats (R820W) has also been identified and was predicted to cause disruptions in the secondary structures and increased hydrophobicity of C6 (Meurs et al., 2007). The R820W substitution has since been found in a patient with HCM and LV non-compaction in 2010 (Ripoll Vera et al., 2010), and shown to have an impact on calcium sensitivity (Messer et al., 2017).

There is also emerging evidence that pathogenic variants that do not alter structure or stability may perturb the nanomechanical features of MyBPC. Single-molecule atomic force spectroscopy experiments showed that polyproteins containing R495W and R502Q substitutions in the C3 domain were mechanically weaker and unfolded faster than other nonpathogenic variants (Suay-Corredera et al., 2021b). It is likely that the C3 domain imposes an internal load to actomyosin sliding, but the R495W and R502Q substitutions may cause a defect in the braking function of MyBPC, leading to accelerated crossbridge cycling and eventual hypercontractility (Suay-Corredera and Alegre-Cebollada, 2022).

In more recent times, it has been possible to study variants of MyBPC with uncertain significance (VUS) and characterize how those variants may impact MyBPC function. Using bioinformatic prediction tools and functional studies (RNA splicing, circular dichroism, and differential scanning calorimetry), it is possible to clarify or even reclassify VUS as pathogenic vs. benign. For example, I603M VUS in domain C4 was shown to cause structural instability which likely impacts function (Pricolo et al., 2020). Although it is necessary to conduct further studies to determine whether this variant has a direct impact on MyBPC function, the authors argue that the reclassification of the I603M substitution as pathogenic may be warranted (Pricolo et al., 2020). As described above, the central domain seems to contain many pathogenic and potentially pathogenic substitutions that impact MyBPC function in diverse ways.

## Post-translational modifications in the central domains of MyBPC

Soon after the discovery of MyBPC, there has been much interest in phosphorylation and its effects on contractility (Jeacocke and England, 1980; Hartzell, 1984; 1985; Hartzell and Glass, 1984; Lim et al., 1985; Weisberg and Winegrad, 1996). Since then, an extensive number of PTMs, including phosphorylation, acetylation, citrullination, S-glutathionylation, S-nitrosylation, and carbonylation, were discovered and are reviewed in many articles (Sadayappan and de Tombe, 2014; Carrier et al., 2015; Main et al., 2020). Here, we provide an overview of PTMs specifically located in the central domains that may have functional importance.

A liquid chromatography-tandem mass spectrometry (LC-MS/MS) study found that cardiac MyBPC residues T601 and T606 in the C4 domain and S707 in the C5 domain were significantly less phosphorylated in failing human hearts compared to healthy controls (Kooij et al., 2013). Another study showed that mice treated with beta-adrenergic receptor antagonists had hyper-phosphorylated MyBPC residues, some within the central domain (Lundby et al., 2013). In a targeted *in vitro* MS study of the C4-C5 domains, ten additional phosphorylation sites were identified within the C4 and C5 domains, including in the C5 28 amino acid loop region (Doh et al., 2022b). These PKA and PKG1 targets within C4-C5 domains were hypothesized by the authors to play a role in intra- and inter-molecular interactions within the sarcomere or implicated in regulation (Doh et al., 2022a; Doh et al., 2022b). Other studies have also identified various phosphorylation sites in the central domains C3-C6 (Schumacher et al., 2007; Huttlin et al., 2010; Hornbeck et al., 2012). Interestingly, the SRX state has been shown to be affected by the phosphorylation status of the N-terminal of MyBPC, but it is unknown whether the phosphorylation of the central domains is also involved (McNamara et al., 2019).

Myofibrils treated with S-nitrosocysteine and/or S-nitrosoglutathione resulted in decreased Ca^2+^ sensitivity due to increased levels of nitrosylation on cysteine residues of several sarcomeric proteins, including C715 in the central domain of MyBPC (Figueiredo-Freitas et al., 2015). Ca^2+^-desensitization from S-nitrosylation significantly decreased maximal force development and an overall reduction in the rate of relaxation (Figueiredo-Freitas et al., 2015). Although many proteins contain nitrosylation sites, the authors suggested that nitrosylation of myofilament proteins generally prevents irreversible protein oxidation, which has downstream cardioprotective effects (Figueiredo-Freitas et al., 2015).

Another oxidative modification, S-glutathionylation, was discovered in MyBPC (Brennan et al., 2006; Lovelock et al., 2012). Tandem mass spectrometry on myofibrils treated with oxidized glutathione showed that MyBPC was S-glutathionylated exclusively in the central domain at residues C479 (C3 domain), and C627 (C4 domain), and C655 (C5 domain) (Patel et al., 2013). They found that S-glutathionylation had a reversible increase in Ca^2+^ sensitivity and ATPase rate, potentially from the maintenance of longitudinal rigidity interactions with cross-bridge constituents (Patel et al., 2013). S-glutathionylation was also associated with *in vivo* diastolic dysfunction, possibly because it prevents radial disposition of MyBPC in order to enter the cross-bridge cycle or alters N-terminal interactions with myosin or actin (Jeong et al., 2013). No matter what the precise mechanism of action, it is clear that the central domain is a hot spot for S-glutathionylation in MyBPC and likely has a prominent effect on function (Jeong et al., 2013; Liu et al., 2021). More recently, it has even been proposed that S-glutathionylated MyBPC could be a potential biomarker for diastolic dysfunction (Zhou et al., 2022a). Full length cardiac MyBPC was immunoprecipitated from blood samples taken from humans, African green monkeys, and mice with confirmed diastolic dysfunction. Circulating levels of S-glutathionylated cardiac MyBPC were positively correlated with diastolic dysfunction in humans and significantly increased in the animal models of diastolic dysfunction compared to disease-free controls (Zhou et al., 2022a). This implicates the clinical relevance of the central domains of MyBPC and its potential for detecting the early stages of heart failure with preserved ejection fraction (HFpEF) and disease stratification (Rosas and Solaro, 2022).

Acetylation, typically regulated by histone acetyltransferase and deacetylase, was first observed in MyBPC near the N-terminal region (Ge et al., 2009; Govindan et al., 2012). Later on, an *in vitro* MS study identified six additional sites of acetylation in the central C4-C5 domains (Doh et al., 2022b). The role of acetylation in MyBPC is thus still unknown but a change from positively to neutrally charged moiety is likely to lead to alterations of surface electrostatic charges and changes in protein interactions.

The only known citrullination in MyBPC was first observed to occur on residue R696 in domain C5 of MyBPC in both healthy and diseased hearts (Fert-Bober and Sokolove, 2014; Fert-Bober et al., 2015). Because citrullination results in loss of charge, similar to acetylation, the authors noted that this change likely causes alterations in protein structure, interactions, proteolytic susceptibility, and intracellular signaling (Fert-Bober et al., 2015). However, additional mutagenesis experiments or *in vivo* studies are necessary to confirm the role of citrullination in MyBPC physiology.

Ubiquitination in the central domains of MyBPC has also been reported, so it can be hypothesized that the central domain is likely targeted for ubiquitination mediated MyBPC degradation (Hornbeck et al., 2012; Wagner et al., 2012; Doh et al., 2022b). A general overview of the mechanisms of ubiquitination in cardiac disease is reviewed elsewhere (Mearini et al., 2008).

Although the roles of many PTMs in the central domains of MyBPC are unknown, these PTMs are likely functionally and clinically significant based on the current review of the literature. In addition, many of the PTM seem to engage in a combinatorial “PTM crosstalk” (Parker et al., 2014; Irie et al., 2015; Stathopoulou et al., 2016; Chakouri et al., 2018; Habibian and Ferguson, 2018; Leutert et al., 2021). As such, future efforts to uncover the ways PTMs influence MyBPC should consider the contributions and actions of simultaneously occurring PTMs.

## Discussion

In this review, we provided a brief overview of the evolution in our understanding of the central domains (C3-C6) of MyBPC. The central domain contains unique structural and dynamic aspects that allow for the formation of IHM via myosin S1 and proximal S2. Thus, the central domains likely impact the SRX state, calcium-activated force generation, contractility, crossbridge recruitment, and *in vivo* function. The above theorized roles of the central domains are further modulated by various potential protein interactions as well as PTMs that may affect IHM formation or the SRX state. Finally, the mutations affecting the central domains’ surface characteristics, folding, or interactions may lead to HCM and contribute to its pathophysiology.

Despite the limited resolution, recent studies employing cryo-EM and cryo-ET have revealed distinct myosin crown arrangements in the relaxed state of myosin filaments, facilitated by the use of mavacamten. These findings mark a substantial leap forward in our comprehension of myosin filament structure, particularly the global positioning of MyBPC with respect to the C-zone. It should be highlighted, though, that in these investigations, the myosin heads are uniformly observed in a folded-back configuration. Future research must investigate the prevalence of this conformation and assess whether all myosin proteins consistently adopt this state or if some exhibit intermediate forms within the relaxed thick filament. Uncovering this will contribute to a deeper and clearer understanding of MyBPC domain positioning within thick filaments. Additionally, delineating the conformations of MyBPC in its native state across different muscle functional states is crucial.

There are many potential avenues for continued research that may help better our knowledge of the central region of MyBPC. Although isolated domain structures can now be readily predicted using *in silico* programs, we still lack a three-dimensional understanding of how the central domain interacts with myosin. Thus, there is an urgent need for high-resolution structures of full-length human cardiac MyBPC. This knowledge is crucial for understanding the molecular mechanisms underlying various cardiac diseases and for developing targeted therapeutic strategies. Finally, the continually expanding and evolving landscape of mutations and PTMs offers a glimpse into the function and regulation of the central domains of MyBPC. Specifically, it is unclear whether PTMs of central domains modulate SRX states or other cross-bridge kinetic parameters. Further research in these areas may help formulate a more complete and comprehensive understanding of the role of MyBPC in cardiac physiology and pathophysiology.

## Abbreviations

MyBPC, myosin binding protein C; HCM, hypertrophic cardiomyopathy; NMR, nuclear magnetic resonance; cryo-EM, cryo-Electron Microscopy; ET, electron tomography; IHM, interacting heads motif; HMM, heavy meromyosin; MST, microscale thermophoresis; SRX, super-relaxed state; DRX, disordered-relaxed state; Y2H, yeast two-hybrid; mECT, murine engineered cardiac tissue; LC-MS/MS, liquid chromatography-tandem mass spectrometry; PTM, post-translational modifications.

## References

[B1] Ackermann M. A. Kontrogianni-Konstantopoulos A. (2011). Myosin binding protein-C: a regulator of actomyosin interaction in striated muscle. J. Biomed. Biotechnol. 2011, 636403. 10.1155/2011/636403 22028592 PMC3196898

[B2] Alamo L. Qi D. Wriggers W. Pinto A. Zhu J. Bilbao A. (2016). Conserved intramolecular interactions maintain myosin interacting-heads motifs explaining tarantula muscle super-relaxed state structural basis. J. Mol. Biol. 428 (6), 1142–1164. 10.1016/j.jmb.2016.01.027 26851071 PMC4826325

[B3] Al-Khayat H. A. Kensler R. W. Squire J. M. Marston S. B. Morris E. P. (2013). Atomic model of the human cardiac muscle myosin filament. Proc. Natl. Acad. Sci. 110 (1), 318–323. 10.1073/pnas.1212708110 23251030 PMC3538242

[B4] Anderson R. L. Trivedi D. V. Sarkar S. S. Henze M. Ma W. Gong H. (2018a). Deciphering the super relaxed state of human β-cardiac myosin and the mode of action of mavacamten from myosin molecules to muscle fibers. Proc. Natl. Acad. Sci. U. S. A. 115 (35), E8143–E8152. 10.1073/pnas.1809540115 30104387 PMC6126717

[B5] Anderson R. L. Trivedi D. V. Sarkar S. S. Henze M. Ma W. Gong H. (2018b). Deciphering the super relaxed state of human β-cardiac myosin and the mode of action of mavacamten from myosin molecules to muscle fibers. Proc. Natl. Acad. Sci. 115 (35), E8143–E8152. 10.1073/pnas.1809540115 30104387 PMC6126717

[B6] Barefield D. Sadayappan S. (2010). Phosphorylation and function of cardiac myosin binding protein-C in health and disease. J. Mol. Cell. Cardiol. 48 (5), 866–875. 10.1016/j.yjmcc.2009.11.014 19962384 PMC6800196

[B7] Belknap B. Harris S. P. White H. D. (2014). Modulation of thin filament activation of myosin ATP hydrolysis by N-terminal domains of cardiac myosin binding protein-C. Biochemistry 53 (42), 6717–6724. 10.1021/bi500787f 25265574 PMC4211651

[B8] Bennett P. M. Fürst D. O. Gautel M. (1999). The C-protein (myosin binding protein C) family: regulators of contraction and sarcomere formation? Rev. Physiology, Biochem. Pharmacol. 138, 203–234. 10.1007/BFb0119628 10396142

[B9] Brennan J. P. Miller J. I. A. Fuller W. Wait R. Begum S. Dunn M. J. (2006). The utility of N,N-biotinyl glutathione disulfide in the study of protein S-glutathiolation. Mol. Cell. Proteomics MCP 5 (2), 215–225. 10.1074/mcp.M500212-MCP200 16223748

[B10] Callaway J. E. Bechtel P. J. (1981). C-protein from rabbit soleus (red) muscle. Biochem. J. 195 (2), 463–469. 10.1042/bj1950463 6797400 PMC1162910

[B11] Carrier L. Bonne G. Bährend E. Yu B. Richard P. Niel F. (1997). Organization and sequence of human cardiac myosin binding protein C gene (MYBPC3) and identification of mutations predicted to produce truncated proteins in familial hypertrophic cardiomyopathy. Circulation Res. 80 (3), 427–434. 10.1161/01.res.0000435859.24609.b3 9048664

[B12] Carrier L. Hengstenberg C. Beckmann J. S. Guicheney P. Dufour C. Bercovici J. (1993). Mapping of a novel gene for familial hypertrophic cardiomyopathy to chromosome 11. Nat. Genet. 4 (3), 311–313. 10.1038/ng0793-311 8358441

[B13] Carrier L. Mearini G. Stathopoulou K. Cuello F. (2015). Cardiac myosin-binding protein C (MYBPC3) in cardiac pathophysiology. Gene 573 (2), 188–197. 10.1016/j.gene.2015.09.008 26358504 PMC6660134

[B14] Carrier L. Schlossarek S. Willis M. S. Eschenhagen T. (2010). The ubiquitin-proteasome system and nonsense-mediated mRNA decay in hypertrophic cardiomyopathy. Cardiovasc. Res. 85 (2), 330–338. 10.1093/cvr/cvp247 19617224 PMC4023315

[B15] Cecconi F. Guardiani C. Livi R. (2008). Analyzing pathogenic mutations of C5 domain from cardiac myosin binding protein C through MD simulations. Eur. Biophysics J. EBJ 37 (5), 683–691. 10.1007/s00249-008-0308-x 18379775

[B16] Chakouri N. Reboul C. Boulghobra D. Kleindienst A. Nottin S. Gayrard S. (2018). Stress-induced protein S-glutathionylation and phosphorylation crosstalk in cardiac sarcomeric proteins - impact on heart function. Int. J. Cardiol. 258, 207–216. 10.1016/j.ijcard.2017.12.004 29544934

[B17] Colson B. A. Thompson A. R. Espinoza-Fonseca L. M. Thomas D. D. (2016). Site-directed spectroscopy of cardiac myosin-binding protein C reveals effects of phosphorylation on protein structural dynamics. Proc. Natl. Acad. Sci. 113 (12), 3233–3238. 10.1073/pnas.1521281113 26908877 PMC4812748

[B18] Desai D. A. Rao V. J. Jegga A. G. Dhandapany P. S. Sadayappan S. (2022). Heterogeneous distribution of genetic mutations in myosin binding protein-C paralogs. Front. Genet. 13, 896117. 10.3389/fgene.2022.896117 35832193 PMC9272480

[B19] Doh C. Y. Bharambe N. Holmes J. B. Dominic K. L. Swanberg C. E. Mamidi R. (2022a). Molecular characterization of linker and loop-mediated structural modulation and hinge motion in the C4-C5 domains of cMyBPC. J. Struct. Biol. 214 (2), 107856. 10.1016/j.jsb.2022.107856 35427781 PMC9942529

[B20] Doh C. Y. Dominic K. L. Swanberg C. E. Bharambe N. Willard B. B. Li L. (2022b). Identification of phosphorylation and other post-translational modifications in the central C4C5 domains of murine cardiac myosin binding protein C. ACS Omega 7 (16), 14189–14202. 10.1021/acsomega.2c00799 35573219 PMC9089392

[B21] Doh C. Y. Kampourakis T. Campbell K. S. Stelzer J. E. (2023). Basic science methods for the characterization of variants of uncertain significance in hypertrophic cardiomyopathy. Front. Cardiovasc. Med. 10, 1238515. 10.3389/fcvm.2023.1238515 37600050 PMC10432852

[B22] Dominic K. L. Choi J. Holmes J. B. Singh M. Majcher M. J. Stelzer J. E. (2023). The contribution of N-terminal truncated cMyBPC to *in vivo* cardiac function. J. General Physiology 155 (6), e202213318. 10.1085/jgp.202213318 PMC10114924 37067542

[B23] Dutta D. Nguyen V. Campbell K. S. Padrón R. Craig R. (2023). Cryo-EM structure of the human cardiac myosin filament. Nature 2023, 853–862. 10.1038/s41586-023-06691-4 PMC10846670 37914935

[B24] Einheber S. Fischman D. A. (1990). Isolation and characterization of a cDNA clone encoding avian skeletal muscle C-protein: an intracellular member of the immunoglobulin superfamily. Proc. Natl. Acad. Sci. U. S. A. 87 (6), 2157–2161. 10.1073/pnas.87.6.2157 2315308 PMC53645

[B25] Fert-Bober J. Giles J. T. Holewinski R. J. Kirk J. A. Uhrigshardt H. Crowgey E. L. (2015). Citrullination of myofilament proteins in heart failure. Cardiovasc. Res. 108 (2), 232–242. 10.1093/cvr/cvv185 26113265 PMC4614685

[B26] Fert-Bober J. Sokolove J. (2014). Proteomics of citrullination in cardiovascular disease. PROTEOMICS - Clin. Appl. 8 (7–8), 522–533. 10.1002/prca.201400013 24946285

[B27] Figueiredo-Freitas C. Dulce R. A. Foster M. W. Liang J. Yamashita A. M. S. Lima-Rosa F. L. (2015). S-nitrosylation of sarcomeric proteins depresses myofilament Ca2+ sensitivity in intact cardiomyocytes. Antioxidants Redox Signal. 23 (13), 1017–1034. 10.1089/ars.2015.6275 PMC4649751 26421519

[B28] Flashman E. Korkie L. Watkins H. Redwood C. Moolman-Smook J. C. (2008). Support for a trimeric collar of myosin binding protein C in cardiac and fast skeletal muscle, but not in slow skeletal muscle. FEBS Lett. 582 (3), 434–438. 10.1016/j.febslet.2008.01.004 18201573

[B29] Flashman E. Redwood C. Moolman-Smook J. Watkins H. (2004). Cardiac myosin binding protein C: its role in physiology and disease. Circulation Res. 94 (10), 1279–1289. 10.1161/01.RES.0000127175.21818.C2 15166115

[B30] Flashman E. Watkins H. Redwood C. (2007). Localization of the binding site of the C-terminal domain of cardiac myosin-binding protein-C on the myosin rod. Biochem. J. 401 (1), 97–102. 10.1042/BJ20060500 16918501 PMC1698665

[B31] Flavigny J. Robert P. Camelin J.-C. Schwartz K. Carrier L. Berrebi-Bertrand I. (2003). Biomolecular interactions between human recombinant beta-MyHC and cMyBP-Cs implicated in familial hypertrophic cardiomyopathy. Cardiovasc. Res. 60 (2), 388–396. 10.1016/j.cardiores.2003.07.001 14613868

[B32] Freiburg A. Gautel M. (1996). A molecular map of the interactions between titin and myosin-binding protein C. Implications for sarcomeric assembly in familial hypertrophic cardiomyopathy. Eur. J. Biochem. 235 (1–2), 317–323. 10.1111/j.1432-1033.1996.00317.x 8631348

[B33] Fürst D. O. Vinkemeier U. Weber K. (1992). Mammalian skeletal muscle C-protein: purification from bovine muscle, binding to titin and the characterization of a full-length human cDNA. J. Cell Sci. 102 (4), 769–778. 10.1242/jcs.102.4.769 1429890

[B34] Gautel M. Zuffardi O. Freiburg A. Labeit S. (1995). Phosphorylation switches specific for the cardiac isoform of myosin binding protein-C: a modulator of cardiac contraction? EMBO J. 14 (9), 1952–1960. 10.1002/j.1460-2075.1995.tb07187.x 7744002 PMC398294

[B35] Ge Y. Rybakova I. N. Xu Q. Moss R. L. (2009). Top-down high-resolution mass spectrometry of cardiac myosin binding protein C revealed that truncation alters protein phosphorylation state. Proc. Natl. Acad. Sci. 106 (31), 12658–12663. 10.1073/pnas.0813369106 19541641 PMC2722289

[B36] Gilbert R. Kelly M. G. Mikawa T. Fischman D. A. (1996). The carboxyl terminus of myosin binding protein C (MyBP-C, C-protein) specifies incorporation into the A-band of striated muscle. J. Cell Sci. 109 (1), 101–111. 10.1242/jcs.109.1.101 8834795

[B37] Glazier A. A. Thompson A. Day S. M. (2019). Allelic imbalance and haploinsufficiency in MYBPC3-linked hypertrophic cardiomyopathy. Pflugers Archiv Eur. J. Physiology 471 (5), 781–793. 10.1007/s00424-018-2226-9 30456444 PMC6476680

[B38] Gollapudi S. K. Yu M. Gan Q.-F. Nag S. (2021). Synthetic thick filaments: a new avenue for better understanding the myosin super-relaxed state in healthy, diseased, and mavacamten-treated cardiac systems. J. Biol. Chem. 296, 100114. 10.1074/jbc.RA120.016506 33234590 PMC7948491

[B39] Govindan S. Sarkey J. Ji X. Sundaresan N. R. Gupta M. P. de Tombe P. P. (2012). Pathogenic properties of the N-terminal region of cardiac myosin binding protein-C *in vitro* . J. Muscle Res. Cell Motil. 33 (1), 17–30. 10.1007/s10974-012-9292-y 22527638 PMC3368277

[B40] Gruen M. Gautel M. (1999). Mutations in beta-myosin S2 that cause familial hypertrophic cardiomyopathy (FHC) abolish the interaction with the regulatory domain of myosin-binding protein-C. J. Mol. Biol. 286 (3), 933–949. 10.1006/jmbi.1998.2522 10024460

[B41] Guardiani C. Cecconi F. Livi R. (2008). Computational analysis of folding and mutation properties of C5 domain of myosin binding protein C. Proteins 70 (4), 1313–1322. 10.1002/prot.21621 17876814

[B42] Habibian J. Ferguson B. S. (2018). The crosstalk between acetylation and phosphorylation: emerging new roles for HDAC inhibitors in the heart. Int. J. Mol. Sci. 20 (1), 102. 10.3390/ijms20010102 30597863 PMC6337125

[B43] Hanson J. O’Brien E. J. Bennett P. M. (1971). Structure of the myosin-containing filament assembly (A-segment) separated from frog skeletal muscle. J. Mol. Biol. 58 (3), 865–871. 10.1016/0022-2836(71)90045-3 4103927

[B44] Harris S. P. Lyons R. G. Bezold K. L. (2011). In the thick of it: HCM-causing mutations in myosin binding proteins of the thick filament. Circulation Res. 108 (6), 751–764. 10.1161/CIRCRESAHA.110.231670 21415409 PMC3076008

[B45] Harris S. P. Rostkova E. Gautel M. Moss R. L. (2004). Binding of myosin binding protein-C to myosin subfragment S2 affects contractility independent of a tether mechanism. Circulation Res. 95 (9), 930–936. 10.1161/01.RES.0000147312.02673.56 15472117

[B46] Hartzell H. C. (1984). Phosphorylation of C-protein in intact amphibian cardiac muscle. Correlation between 32P incorporation and twitch relaxation. J. General Physiology 83 (4), 563–588. 10.1085/jgp.83.4.563 PMC2215646 6547162

[B47] Hartzell H. C. (1985). Effects of phosphorylated and unphosphorylated C-protein on cardiac actomyosin ATPase. J. Mol. Biol. 186 (1), 185–195. 10.1016/0022-2836(85)90268-2 2934553

[B48] Hartzell H. C. Glass D. B. (1984). Phosphorylation of purified cardiac muscle C-protein by purified cAMP-dependent and endogenous Ca2+-calmodulin-dependent protein kinases. J. Biol. Chem. 259 (24), 15587–15596. 10.1016/s0021-9258(17)42588-9 6549009

[B49] Hartzell H. C. Sale W. S. (1985). Structure of C protein purified from cardiac muscle. J. Cell Biol. 100 (1), 208–215. 10.1083/jcb.100.1.208 3838095 PMC2113476

[B50] Heissler S. M. Arora A. S. Billington N. Sellers J. R. Chinthalapudi K. (2021). Cryo-EM structure of the autoinhibited state of myosin-2. Sci. Adv. 7 (52), eabk3273. 10.1126/sciadv.abk3273 34936462 PMC8694606

[B51] Heling L. W. H. J. Geeves M. A. Kad N. M. (2020). MyBP-C: one protein to govern them all. J. Muscle Res. Cell Motil. 41 (1), 91–101. 10.1007/s10974-019-09567-1 31960266 PMC7109175

[B52] Helms A. S. Thompson A. D. Glazier A. A. Hafeez N. Kabani S. Rodriguez J. (2020). Spatial and functional distribution of *MYBPC3* pathogenic variants and clinical outcomes in patients with hypertrophic cardiomyopathy. Circulation Genomic Precis. Med. 13 (5), 396–405. 10.1161/CIRCGEN.120.002929 PMC7676622 32841044

[B53] Hofmann P. A. Hartzell H. C. Moss R. L. (1991). Alterations in Ca2+ sensitive tension due to partial extraction of C-protein from rat skinned cardiac myocytes and rabbit skeletal muscle fibers. J. General Physiology 97 (6), 1141–1163. 10.1085/jgp.97.6.1141 PMC2216516 1678777

[B54] Hooijman P. Stewart M. A. Cooke R. (2011). A new state of cardiac myosin with very slow ATP turnover: a potential cardioprotective mechanism in the heart. Biophysical J. 100 (8), 1969–1976. 10.1016/j.bpj.2011.02.061 PMC3077696 21504733

[B55] Hornbeck P. V. Kornhauser J. M. Tkachev S. Zhang B. Skrzypek E. Murray B. (2012). PhosphoSitePlus: a comprehensive resource for investigating the structure and function of experimentally determined post-translational modifications in man and mouse. Nucleic Acids Res. 40 (D1), D261–D270. 10.1093/nar/gkr1122 22135298 PMC3245126

[B56] Huttlin E. L. Jedrychowski M. P. Elias J. E. Goswami T. Rad R. Beausoleil S. A. (2010). A tissue-specific atlas of mouse protein phosphorylation and expression. Cell 143 (7), 1174–1189. 10.1016/j.cell.2010.12.001 21183079 PMC3035969

[B57] Huxley H. E. (1967). Recent x-ray diffraction and electron microscope studies of striated muscle. J. General Physiology 50 (6), 71–83. 10.1085/jgp.50.6.71 PMC2225753 4227925

[B58] Huxley H. E. Brown W. (1967). The low-angle x-ray diagram of vertebrate striated muscle and its behaviour during contraction and rigor. J. Mol. Biol. 30 (2), 383–434. 10.1016/s0022-2836(67)80046-9 5586931

[B59] Idowu S. M. Gautel M. Perkins S. J. Pfuhl M. (2003). Structure, stability and dynamics of the central domain of cardiac myosin binding protein C (MyBP-C): implications for multidomain assembly and causes for cardiomyopathy. J. Mol. Biol. 329 (4), 745–761. 10.1016/S0022-2836(03)00425-X 12787675

[B60] Irie T. Sips P. Y. Kai S. Kida K. Ikeda K. Hirai S. (2015). S-nitrosylation of calcium-handling proteins in cardiac adrenergic signaling and hypertrophy. Circulation Res. 117 (9), 793–803. 10.1161/CIRCRESAHA.115.307157 26259881 PMC4600453

[B61] Jeacocke S. A. England P. J. (1980). Phosphorylation of a myofibrillar protein of Mr 150 000 in perfused rat heart, and the tentative indentification of this as C-protein. FEBS Lett. 122 (1), 129–132. 10.1016/0014-5793(80)80418-2 6260526

[B62] Jeong E.-M. Monasky M. M. Gu L. Taglieri D. M. Patel B. G. Liu H. (2013). Tetrahydrobiopterin improves diastolic dysfunction by reversing changes in myofilament properties. J. Mol. Cell. Cardiol. 56, 44–54. 10.1016/j.yjmcc.2012.12.003 23247392 PMC3666585

[B63] Jumper J. Evans R. Pritzel A. Green T. Figurnov M. Ronneberger O. (2021). Highly accurate protein structure prediction with AlphaFold. Nature 596 (7873), 583–589. 10.1038/s41586-021-03819-2 34265844 PMC8371605

[B64] Karsai Á. Kellermayer M. S. Z. Harris S. P. (2013). Cross-species mechanical fingerprinting of cardiac myosin binding protein-C. Biophysical J. 104 (11), 2465–2475. 10.1016/j.bpj.2013.04.027 PMC3672900 23746519

[B65] Kawashima M. Kitani S. Tanaka T. Obinata T. (1986). The earliest form of C-protein expressed during striated muscle development is immunologically the same as cardiac-type C-protein. J. Biochem. 99 (4), 1037–1047. 10.1093/oxfordjournals.jbchem.a135567 3519599

[B66] Kensler R. W. Shaffer J. F. Harris S. P. (2011). Binding of the N-terminal fragment C0-C2 of cardiac MyBP-C to cardiac F-actin. J. Struct. Biol. 174 (1), 44–51. 10.1016/j.jsb.2010.12.003 21163356 PMC3056911

[B67] Kooij V. Holewinski R. J. Murphy A. M. Van Eyk J. E. (2013). Characterization of the cardiac myosin binding protein-C phosphoproteome in healthy and failing human hearts. J. Mol. Cell. Cardiol. 60 (1), 116–120. 10.1016/j.yjmcc.2013.04.012 23619294 PMC3710717

[B68] Lee K. Harris S. P. Sadayappan S. Craig R. (2015). Orientation of myosin binding protein C in the cardiac muscle sarcomere determined by domain-specific immuno-EM. J. Mol. Biol. 427 (2), 274–286. 10.1016/j.jmb.2014.10.023 25451032 PMC4297556

[B69] Leutert M. Entwisle S. W. Villén J. (2021). Decoding post-translational modification crosstalk with proteomics. Mol. Cell. Proteomics MCP 20, 100129. 10.1016/j.mcpro.2021.100129 34339852 PMC8430371

[B70] Li J. Mamidi R. Doh C. Y. Holmes J. B. Bharambe N. Ramachandran R. (2020). AAV9 gene transfer of cMyBPC N-terminal domains ameliorates cardiomyopathy in cMyBPC-deficient mice. JCI Insight 5 (17), e130182. 10.1172/jci.insight.130182 32750038 PMC7526450

[B71] Lim M. S. Sutherland C. Walsh M. P. (1985). Phosphorylation of bovine cardiac C-protein by protein kinase C. Biochem. Biophysical Res. Commun. 132 (3), 1187–1195. 10.1016/0006-291x(85)91932-1 3840998

[B72] Liu M. Liu H. Feng F. Xie A. Kang G.-J. Zhao Y. (2021). Magnesium deficiency causes a reversible, metabolic, diastolic cardiomyopathy. J. Am. Heart Assoc. 10 (12), e020205. 10.1161/JAHA.120.020205 34096318 PMC8477865

[B73] Lovelock J. D. Monasky M. M. Jeong E.-M. Lardin H. A. Liu H. Patel B. G. (2012). Ranolazine improves cardiac diastolic dysfunction through modulation of myofilament calcium sensitivity. Circulation Res. 110 (6), 841–850. 10.1161/CIRCRESAHA.111.258251 22343711 PMC3314887

[B74] Lu Y. Kwan A. H. Jeffries C. M. Guss J. M. Trewhella J. (2012). The motif of human cardiac myosin-binding protein C is required for its Ca2+-dependent interaction with calmodulin. J. Biol. Chem. 287 (37), 31596–31607. 10.1074/jbc.M112.383299 22801425 PMC3438991

[B75] Lundby A. Andersen M. N. Steffensen A. B. Horn H. Kelstrup C. D. Francavilla C. (2013). *In vivo* phosphoproteomics analysis reveals the cardiac targets of β-adrenergic receptor signaling. Sci. Signal. 6 (278), rs11–14. 10.1126/scisignal.2003506 23737553

[B76] Luther P. K. Winkler H. Taylor K. Zoghbi M. E. Craig R. Padrón R. (2011). Direct visualization of myosin-binding protein C bridging myosin and actin filaments in intact muscle. Proc. Natl. Acad. Sci. U. S. A. 108 (28), 11423–11428. 10.1073/pnas.1103216108 21705660 PMC3136262

[B77] Lynch T. L. Kumar M. McNamara J. W. Kuster D. W. D. Sivaguru M. Singh R. R. (2021). Amino terminus of cardiac myosin binding protein-C regulates cardiac contractility. J. Mol. Cell. Cardiol. 156, 33–44. 10.1016/j.yjmcc.2021.03.009 33781820 PMC8217138

[B78] Main A. Fuller W. Baillie G. S. (2020). Post-translational regulation of cardiac myosin binding protein-C: a graphical review. Cell. Signal. 76, 109788. 10.1016/j.cellsig.2020.109788 32976931

[B79] Mamidi R. Gresham K. S. Verma S. Stelzer J. E. (2016). Cardiac myosin binding protein-C phosphorylation modulates myofilament length-dependent activation. Front. Physiology 7, 38. 10.3389/fphys.2016.00038 PMC4753332 26913007

[B80] Maron B. J. Maron M. S. (2013). Hypertrophic cardiomyopathy. Lancet 381 (9862), 242–255. 10.1016/S0140-6736(12)60397-3 22874472

[B81] Matsuyama S. Kage Y. Fujimoto N. Ushijima T. Tsuruda T. Kitamura K. (2018). Interaction between cardiac myosin-binding protein C and formin Fhod3. Proc. Natl. Acad. Sci. U. S. A. 115 (19), E4386–E4395. 10.1073/pnas.1716498115 29686099 PMC5948958

[B82] McClellan G. Kulikovskaya I. Flavigny J. Carrier L. Winegrad S. (2004). Effect of cardiac myosin-binding protein C on stability of the thick filament. J. Mol. Cell. Cardiol. 37 (4), 823–835. 10.1016/j.yjmcc.2004.05.023 15380673

[B83] McGrath M. J. Cottle D. L. Nguyen M.-A. Dyson J. M. Coghill I. D. Robinson P. A. (2006a). Four and a half LIM protein 1 binds myosin-binding protein C and regulates myosin filament formation and sarcomere assembly. J. Biol. Chem. 281 (11), 7666–7683. 10.1074/jbc.M512552200 16407297

[B84] McGrath M. J. Cottle D. L. Nguyen M.-A. Dyson J. M. Coghill I. D. Robinson P. A. (2006b). Four and a half LIM protein 1 binds myosin-binding protein C and regulates myosin filament formation and sarcomere assembly. J. Biol. Chem. 281 (11), 7666–7683. 10.1074/jbc.M512552200 16407297

[B85] McNamara J. W. Li A. Lal S. Bos J. M. Harris S. P. van der Velden J. (2017). MYBPC3 mutations are associated with a reduced super-relaxed state in patients with hypertrophic cardiomyopathy. PloS One 12 (6), e0180064. 10.1371/journal.pone.0180064 28658286 PMC5489194

[B86] McNamara J. W. Sadayappan S. (2018). Skeletal myosin binding protein-C: an increasingly important regulator of striated muscle physiology. Archives Biochem. Biophysics 660, 121–128. 10.1016/j.abb.2018.10.007 PMC6289839 30339776

[B87] McNamara J. W. Singh R. R. Sadayappan S. (2019). Cardiac myosin binding protein-C phosphorylation regulates the super-relaxed state of myosin. Proc. Natl. Acad. Sci. U. S. A. 116 (24), 11731–11736. 10.1073/pnas.1821660116 31142654 PMC6575167

[B88] Mearini G. Schlossarek S. Willis M. S. Carrier L. (2008). The ubiquitin-proteasome system in cardiac dysfunction. Biochimica Biophysica Acta 1782 (12), 749–763. 10.1016/j.bbadis.2008.06.009 18634872

[B89] Merkulov S. Chen X. Chandler M. P. Stelzer J. E. (2012). *In vivo* cardiac myosin binding protein C gene transfer rescues myofilament contractile dysfunction in cardiac myosin binding protein C null mice. Circ. Heart Fail. 5 (5), 635–644. 10.1161/CIRCHEARTFAILURE.112.968941 22855556 PMC4860813

[B90] Messer A. E. Chan J. Daley A. Copeland O. Marston S. B. Connolly D. J. (2017). Investigations into the sarcomeric protein and Ca2+-regulation abnormalities underlying hypertrophic cardiomyopathy in cats (felix catus). Front. Physiology 8, 348. 10.3389/fphys.2017.00348 PMC5462916 28642712

[B91] Meurs K. M. Norgard M. M. Ederer M. M. Hendrix K. P. Kittleson M. D. (2007). A substitution mutation in the myosin binding protein C gene in ragdoll hypertrophic cardiomyopathy. Genomics 90 (2), 261–264. 10.1016/j.ygeno.2007.04.007 17521870

[B92] Moolman-Smook J. Flashman E. de Lange W. Li Z. Corfield V. Redwood C. (2002). Identification of novel interactions between domains of Myosin binding protein-C that are modulated by hypertrophic cardiomyopathy missense mutations. Circulation Res. 91 (8), 704–711. 10.1161/01.res.0000036750.81083.83 12386147

[B93] Moos C. Feng I. N. (1980). Effect of C-protein on actomyosin ATPase. Biochimica Biophysica Acta 632 (2), 141–149. 10.1016/0304-4165(80)90071-9 6448079

[B94] Moos C. Mason C. M. Besterman J. M. Feng I. N. Dubin J. H. (1978). The binding of skeletal muscle C-protein to F-actin, and its relation to the interaction of actin with myosin subfragment-1. J. Mol. Biol. 124 (4), 571–586. 10.1016/0022-2836(78)90172-9 152359

[B95] Moos C. Offer G. Starr R. Bennett P. (1975). Interaction of C-protein with myosin, myosin rod and light meromyosin. J. Mol. Biol. 97 (1), 1–9. 10.1016/s0022-2836(75)80017-9 1100851

[B96] Mun J. Y. Gulick J. Robbins J. Woodhead J. Lehman W. Craig R. (2011). Electron microscopy and 3D reconstruction of F-actin decorated with cardiac myosin-binding protein C (cMyBP-C). J. Mol. Biol. 410 (2), 214–225. 10.1016/j.jmb.2011.05.010 21601575 PMC3115431

[B97] Mun J. Y. Previs M. J. Yu H. Y. Gulick J. Tobacman L. S. Previs S. B. (2014). Myosin-binding protein C displaces tropomyosin to activate cardiac thin filaments and governs their speed by an independent mechanism. Proc. Natl. Acad. Sci. U. S. A. 111 (6), 2170–2175. 10.1073/pnas.1316001111 24477690 PMC3926057

[B98] Nadvi N. A. Michie K. A. Kwan A. H. Guss J. M. Trewhella J. (2016). Clinically linked mutations in the central domains of cardiac myosin-binding protein C with distinct phenotypes show differential structural effects. Structure 24 (1), 105–115. 10.1016/j.str.2015.11.001 26688216

[B99] Nag S. Trivedi D. V. (2021). To lie or not to lie: super-relaxing with myosins. ELife 10, e63703. 10.7554/eLife.63703 33565963 PMC7875563

[B100] Nag S. Trivedi D. V. Sarkar S. S. Adhikari A. S. Sunitha M. S. Sutton S. (2017). The myosin mesa and the basis of hypercontractility caused by hypertrophic cardiomyopathy mutations. Nat. Struct. Mol. Biol. 24 (6), 525–533. 10.1038/nsmb.3408 28481356 PMC5737966

[B101] Nogara L. Naber N. Pate E. Canton M. Reggiani C. Cooke R. (2016). Spectroscopic studies of the super relaxed state of skeletal muscle. PloS One 11 (8), e0160100. 10.1371/journal.pone.0160100 27479128 PMC4968846

[B102] Oakley C. E. Hambly B. D. Curmi P. M. G. Brown L. J. (2004). Myosin binding protein C: structural abnormalities in familial hypertrophic cardiomyopathy. Cell Res. 14 (2), 95–110. 10.1038/sj.cr.7290208 15115610

[B103] Offer G. Moos C. Starr R. (1973). A new protein of the thick filaments of vertebrate skeletal myofibrils. Extractions, purification and characterization. J. Mol. Biol. 74 (4), 653–676. 10.1016/0022-2836(73)90055-7 4269687

[B104] Okagaki T. Weber F. E. Fischman D. A. Vaughan K. T. Mikawa T. Reinach F. C. (1993). The major myosin-binding domain of skeletal muscle MyBP-C (C protein) resides in the COOH-terminal, immunoglobulin C2 motif. J. Cell Biol. 123 (3), 619–626. 10.1083/jcb.123.3.619 8227129 PMC2200114

[B105] Orlova A. Galkin V. E. Jeffries C. M. J. Egelman E. H. Trewhella J. (2011). The N-terminal domains of myosin binding protein C can bind polymorphically to F-actin. J. Mol. Biol. 412 (3), 379–386. 10.1016/j.jmb.2011.07.056 21821050 PMC3167005

[B106] Parker B. L. Shepherd N. E. Trefely S. Hoffman N. J. White M. Y. Engholm-Keller K. (2014). Structural basis for phosphorylation and lysine acetylation cross-talk in a kinase motif associated with myocardial ischemia and cardioprotection. J. Biol. Chem. 289 (37), 25890–25906. 10.1074/jbc.M114.556035 25008320 PMC4162189

[B107] Patel B. G. Wilder T. Solaro R. J. (2013). Novel control of cardiac myofilament response to calcium by S-glutathionylation at specific sites of myosin binding protein C. Front. Physiology 4, 336–411. 10.3389/fphys.2013.00336 PMC3834529 24312057

[B108] Pearce A. Ponnam S. Holt M. R. Randall T. Beckingham R. Kho A. L. (2023). Missense mutations in the central domains of cardiac Myosin binding protein-C and their potential contribution to hypertrophic cardiomyopathy. J. Biol. Chem. 105511, 105511. 10.1016/j.jbc.2023.105511 PMC10772716 38042491

[B109] Pepe F. A. Drucker B. (1975). The myosin filament. III. C-protein. J. Mol. Biol. 99 (4), 609–617. 10.1016/s0022-2836(75)80175-6 814246

[B110] Ponnam S. Kampourakis T. (2022). Microscale thermophoresis suggests a new model of regulation of cardiac myosin function via interaction with cardiac myosin-binding protein C. J. Biol. Chem. 298 (1), 101485. 10.1016/j.jbc.2021.101485 34915024 PMC8733265

[B111] Previs M. J. Mun J. Y. Michalek A. J. Beck Previs S. Gulick J. Robbins J. (2016). Phosphorylation and calcium antagonistically tune myosin-binding protein C’s structure and function. Proc. Natl. Acad. Sci. U. S. A. 113 (12), 3239–3244. 10.1073/pnas.1522236113 26908872 PMC4812749

[B112] Pricolo M. R. Herrero-Galán E. Mazzaccara C. Losi M. A. Alegre-Cebollada J. Frisso G. (2020). Protein thermodynamic destabilization in the assessment of pathogenicity of a variant of uncertain significance in cardiac myosin binding protein C. J. Cardiovasc. Transl. Res. 13 (5), 867–877. 10.1007/s12265-020-09959-6 32034629

[B113] Rahmanseresht S. Lee K. H. O’Leary T. S. McNamara J. W. Sadayappan S. Robbins J. (2021). The N terminus of myosin-binding protein C extends toward actin filaments in intact cardiac muscle. J. General Physiology 153 (3), e202012726. 10.1085/jgp.202012726 PMC7852460 33528507

[B114] Ratti J. Rostkova E. Gautel M. Pfuhl M. (2011). Structure and interactions of myosin-binding protein C domain C0: cardiac-specific regulation of myosin at its neck? J. Biol. Chem. 286 (14), 12650–12658. 10.1074/jbc.M110.156646 21297165 PMC3069465

[B115] Razumova M. V. Bezold K. L. Tu A.-Y. Regnier M. Harris S. P. (2008). Contribution of the myosin binding protein C motif to functional effects in permeabilized rat trabeculae. J. General Physiology 132 (5), 575–585. 10.1085/jgp.200810013 PMC2571974 18955596

[B116] Reinach F. C. Masaki T. Shafiq S. Obinata T. Fischman D. A. (1982). Isoforms of C-protein in adult chicken skeletal muscle: detection with monoclonal antibodies. J. Cell Biol. 95 (1), 78–84. 10.1083/jcb.95.1.78 6183271 PMC2112370

[B117] Ripoll Vera T. Monserrat Iglesias L. Hermida Prieto M. Ortiz M. Rodriguez Garcia I. Govea Callizo N. (2010). The R820W mutation in the MYBPC3 gene, associated with hypertrophic cardiomyopathy in cats, causes hypertrophic cardiomyopathy and left ventricular non-compaction in humans. Int. J. Cardiol. 145 (2), 405–407. 10.1016/j.ijcard.2010.04.032 20542340

[B118] Risi C. Belknap B. Forgacs-Lonart E. Harris S. P. Schröder G. F. White H. D. (2018). N-terminal domains of cardiac myosin binding protein C cooperatively activate the thin filament. Structure 26 (12), 1604–1611. 10.1016/j.str.2018.08.007 30270174 PMC6281772

[B119] Rohde J. A. Roopnarine O. Thomas D. D. Muretta J. M. (2018). Mavacamten stabilizes an autoinhibited state of two-headed cardiac myosin. Proc. Natl. Acad. Sci. 115 (32), E7486–E7494. 10.1073/pnas.1720342115 30018063 PMC6094135

[B120] Rome E. Offer G. Pepe F. A. (1973). X-ray diffraction of muscle labelled with antibody to C-protein. Nat. New Biol. 244 (135), 152–154. 10.1038/newbio244152a0 4516378

[B121] Rosas P. C. Liu Y. Abdalla M. I. Thomas C. M. Kidwell D. T. Dusio G. F. (2015). Phosphorylation of cardiac Myosin-binding protein-C is a critical mediator of diastolic function. Circ. Heart Fail. 8 (3), 582–594. 10.1161/CIRCHEARTFAILURE.114.001550 25740839 PMC4447128

[B122] Rosas P. C. Solaro R. J. (2022). Implications of S-glutathionylation of sarcomere proteins in cardiac disorders, therapies, and diagnosis. Front. Cardiovasc. Med. 9, 1060716. 10.3389/fcvm.2022.1060716 36762302 PMC9902711

[B123] Saberi S. Day S. M. (2018). Exercise and hypertrophic cardiomyopathy: time for a change of heart. Circulation 137 (5), 419–421. 10.1161/CIRCULATIONAHA.117.029989 29378753

[B124] Sadayappan S. de Tombe P. P. (2014). Cardiac myosin binding protein-C as a central target of cardiac sarcomere signaling: a special mini review series. Pflügers Archiv - Eur. J. Physiology 466 (2), 195–200. 10.1007/s00424-013-1396-8 24196566 PMC3946865

[B125] Sarkar S. S. Trivedi D. V. Morck M. M. Adhikari A. S. Pasha S. N. Ruppel K. M. (2020). The hypertrophic cardiomyopathy mutations R403Q and R663H increase the number of myosin heads available to interact with actin. Sci. Adv. 6 (14), eaax0069. 10.1126/sciadv.aax0069 32284968 PMC7124958

[B126] Schlender K. K. Bean L. J. (1991). Phosphorylation of chicken cardiac C-protein by calcium/calmodulin-dependent protein kinase II. J. Biol. Chem. 266 (5), 2811–2817. 10.1016/S0021-9258(18)49919-X 1671569

[B127] Schumacher J. A. Crockett D. K. Elenitoba-Johnson K. S. J. Lim M. S. (2007). Evaluation of enrichment techniques for mass spectrometry: identification of tyrosine phosphoproteins in cancer cells. J. Mol. Diagnostics 9 (2), 169–177. 10.2353/jmoldx.2007.060031 PMC1867451 17384208

[B128] Smelter D. F. de Lange W. J. Cai W. Ge Y. Ralphe J. C. (2018). The HCM-linked W792R mutation in cardiac myosin-binding protein C reduces C6 FnIII domain stability. Am. J. Physiology-Heart Circulatory Physiology 314 (6), H1179–H1191. 10.1152/ajpheart.00686.2017 PMC6032085 29451820

[B129] Song T. Landim-Vieira M. Ozdemir M. Gott C. Kanisicak O. Pinto J. R. (2023). Etiology of genetic muscle disorders induced by mutations in fast and slow skeletal MyBP-C paralogs. Exp. Mol. Med. 55 (3), 502–509. 10.1038/s12276-023-00953-x 36854776 PMC10073172

[B130] Squire J. M. Luther P. K. Knupp C. (2003). Structural evidence for the interaction of C-protein (MyBP-C) with actin and sequence identification of a possible actin-binding domain. J. Mol. Biol. 331 (3), 713–724. 10.1016/s0022-2836(03)00781-2 12899839

[B131] Starr R. Offer G. (1971). Polypeptide chains of intermediate molecular weight in myosin preparations. FEBS Lett. 15 (1), 40–44. 10.1016/0014-5793(71)80075-3 11945810

[B132] Starr R. Offer G. (1978). The interaction of C-protein with heavy meromyosin and subfragment-2. Biochem. J. 171 (3), 813–816. 10.1042/bj1710813 352343 PMC1184031

[B133] Stathopoulou K. Wittig I. Heidler J. Piasecki A. Richter F. Diering S. (2016). S-glutathiolation impairs phosphoregulation and function of cardiac myosin-binding protein C in human heart failure. FASEB J. Official Publ. Fed. Am. Soc. Exp. Biol. 30 (5), 1849–1864. 10.1096/fj.201500048 PMC4836368 26839380

[B134] Stewart M. A. Franks-Skiba K. Chen S. Cooke R. (2010). Myosin ATP turnover rate is a mechanism involved in thermogenesis in resting skeletal muscle fibers. Proc. Natl. Acad. Sci. U. S. A. 107 (1), 430–435. 10.1073/pnas.0909468107 19966283 PMC2806748

[B135] Suay-Corredera C. Alegre-Cebollada J. (2022). The mechanics of the heart: zooming in on hypertrophic cardiomyopathy and cMyBP-C. FEBS Lett. 596 (6), 703–746. 10.1002/1873-3468.14301 35224729

[B136] Suay-Corredera C. Pricolo M. R. Herrero-Galán E. Velázquez-Carreras D. Sánchez-Ortiz D. García-Giustiniani D. (2021a). Protein haploinsufficiency drivers identify MYBPC3 variants that cause hypertrophic cardiomyopathy. J. Biol. Chem. 297 (1), 100854. 10.1016/j.jbc.2021.100854 34097875 PMC8260873

[B137] Suay-Corredera C. Pricolo M. R. Velázquez-Carreras D. Pathak D. Nandwani N. Pimenta-Lopes C. (2021b). Nanomechanical phenotypes in cardiac myosin-binding protein C mutants that cause hypertrophic cardiomyopathy. ACS Nano 15 (6), 10203–10216. 10.1021/acsnano.1c02242 34060810 PMC8514129

[B138] Swan R. C. Fischman D. A. (1986). Electron microscopy of C-protein molecules from chicken skeletal muscle. J. Muscle Res. Cell Motil. 7 (2), 160–166. 10.1007/BF01753417 3754879

[B139] Tamborrini D. Wang Z. Wagner T. Tacke S. Stabrin M. Grange M. (2023). Structure of the native myosin filament in the relaxed cardiac sarcomere. Nature 2023, 863–871. 10.1038/s41586-023-06690-5 PMC10665186 37914933

[B140] Thompson A. D. Helms A. S. Kannan A. Yob J. Lakdawala N. K. Wittekind S. G. (2021). Computational prediction of protein subdomain stability in MYBPC3 enables clinical risk stratification in hypertrophic cardiomyopathy and enhances variant interpretation. Genet. Med. 23 (7), 1281–1287. 10.1038/s41436-021-01134-9 33782553 PMC8257482

[B141] Tonino P. Kiss B. Gohlke J. Smith J. E. Granzier H. (2019). Fine mapping titin’s C-zone: matching cardiac myosin-binding protein C stripes with titin’s super-repeats. J. Mol. Cell. Cardiol. 133, 47–56. 10.1016/j.yjmcc.2019.05.026 31158359 PMC6639027

[B142] Tudurachi B.-S. Zăvoi A. Leonte A. Țăpoi L. Ureche C. Bîrgoan S. G. (2023). An update on MYBPC3 gene mutation in hypertrophic cardiomyopathy. Int. J. Mol. Sci. 24 (13), 10510. 10.3390/ijms241310510 37445689 PMC10341819

[B143] Wagner S. A. Beli P. Weinert B. T. Schölz C. Kelstrup C. D. Young C. (2012). Proteomic analyses reveal divergent ubiquitylation site patterns in murine tissues. Mol. Cell. Proteomics 11 (12), 1578–1585. 10.1074/mcp.M112.017905 22790023 PMC3518112

[B144] Walklate J. Kao K. Regnier M. Geeves M. A. (2022). Exploring the super-relaxed state of myosin in myofibrils from fast-twitch, slow-twitch, and cardiac muscle. J. Biol. Chem. 298 (3), 101640. 10.1016/j.jbc.2022.101640 35090895 PMC8867123

[B145] Watkins H. Conner D. Thierfelder L. Jarcho J. A. MacRae C. McKenna W. J. (1995). Mutations in the cardiac myosin binding protein-C gene on chromosome 11 cause familial hypertrophic cardiomyopathy. Nat. Genet. 11 (4), 434–437. 10.1038/ng1295-434 7493025

[B146] Weber F. E. Vaughan K. T. Reinach F. C. Fischman D. A. (1993). Complete sequence of human fast-type and slow-type muscle myosin-binding-protein C (MyBP-C). Differential expression, conserved domain structure and chromosome assignment. Eur. J. Biochem. 216 (2), 661–669. 10.1111/j.1432-1033.1993.tb18186.x 8375400

[B147] Weisberg A. Winegrad S. (1996). Alteration of myosin cross bridges by phosphorylation of myosin-binding protein C in cardiac muscle. Proc. Natl. Acad. Sci. U. S. A. 93 (17), 8999–9003. 10.1073/pnas.93.17.8999 8799143 PMC38584

[B148] Weissler-Snir A. Allan K. Cunningham K. Connelly K. A. Lee D. S. Spears D. A. (2019). Hypertrophic cardiomyopathy-related sudden cardiac death in young people in ontario. Circulation 140 (21), 1706–1716. 10.1161/CIRCULATIONAHA.119.040271 31630535

[B149] Wendt T. Taylor D. Trybus K. M. Taylor K. (2001). Three-dimensional image reconstruction of dephosphorylated smooth muscle heavy meromyosin reveals asymmetry in the interaction between myosin heads and placement of subfragment 2. Proc. Natl. Acad. Sci. U. S. A. 98 (8), 4361–4366. 10.1073/pnas.071051098 11287639 PMC31840

[B150] Whitten A. E. Jeffries C. M. Harris S. P. Trewhella J. (2008). Cardiac myosin-binding protein C decorates F-actin: implications for cardiac function. Proc. Natl. Acad. Sci. U. S. A. 105 (47), 18360–18365. 10.1073/pnas.0808903105 19011110 PMC2587536

[B151] Winkelmann D. A. Forgacs E. Miller M. T. Stock A. M. (2015). Structural basis for drug-induced allosteric changes to human β-cardiac myosin motor activity. Nat. Commun. 6, 7974. 10.1038/ncomms8974 26246073 PMC4918383

[B152] Yamamoto K. Moos C. (1983). The C-proteins of rabbit red, white, and cardiac muscles. J. Biol. Chem. 258 (13), 8395–8401. 10.1016/s0021-9258(20)82078-x 6134729

[B153] Zhang X. L. De S. McIntosh L. P. Paetzel M. (2014). Structural characterization of the C3 domain of cardiac myosin binding protein C and its hypertrophic cardiomyopathy-related R502W mutant. Biochemistry 53 (32), 5332–5342. 10.1021/bi500784g 25058872

[B154] Zhou X. Jeong E.-M. Liu H. Kaseer B. Liu M. Shrestha S. (2022a). Circulating S-glutathionylated cMyBP-C as a biomarker for cardiac diastolic dysfunction. J. Am. Heart Assoc. 11 (11), e025295. 10.1161/JAHA.122.025295 35656993 PMC9238749

[B155] Zhou X. Zheng W. Li Y. Pearce R. Zhang C. Bell E. W. (2022b). I-TASSER-MTD: a deep-learning-based platform for multi-domain protein structure and function prediction. Nat. Protoc. 17 (10), 2326–2353. 10.1038/s41596-022-00728-0 35931779

[B156] Zoghbi M. E. Woodhead J. L. Moss R. L. Craig R. (2008). Three-dimensional structure of vertebrate cardiac muscle myosin filaments. Proc. Natl. Acad. Sci. U. S. A. 105 (7), 2386–2390. 10.1073/pnas.0708912105 18252826 PMC2268146

